# Hyper-Theory-of-Mind in Children with Psychotic Experiences

**DOI:** 10.1371/journal.pone.0113082

**Published:** 2014-11-14

**Authors:** Lars Clemmensen, Jim van Os, Anne Mette Skovgaard, Mette Væver, Els M. A. Blijd-Hoogewys, Agna A. Bartels-Velthuis, Pia Jeppesen

**Affiliations:** 1 Child and Adolescent Mental Health Center, Mental Health Services, the Capital Region of Denmark, Copenhagen, Denmark; 2 Department of Psychiatry and Psychology, Maastricht University Medical Centre, Maastricht, The Netherlands; 3 Department of Public Health, University of Copenhagen, Copenhagen, Denmark; 4 Department of Psychology, University of Copenhagen, Copenhagen, Denmark; 5 INTER-PSY Groningen, Groningen, The Netherlands; 6 University of Groningen, University Medical Center Groningen, University Center for Psychiatry Groningen, Groningen Triadegebouw, The Netherlands; United (Osaka U, Kanazawa U, Hamamatsu U Sch Med, Chiba U and Fukui U) Graduate School of Child Development, Japan

## Abstract

**Background:**

Alterations in Theory-of-Mind (ToM) are associated with psychotic disorder. In addition, studies in children have documented that alterations in ToM are associated with Psychotic Experiences (PE). Our aim was to examine associations between an exaggerated type of ToM (HyperToM) and PE in children. Children with this type of alteration in ToM infer mental states when none are obviously suggested, and predict behaviour on the basis of these erroneous beliefs. Individuals with HyperToM do not appear to have a conceptual deficit (i.e. lack of representational abilities), but rather they apply their theory of the minds of others in an incorrect or biased way.

**Method:**

Hypotheses were tested in two studies with two independent samples: (i) a general population sample of 1630 Danish children aged 11–12 years, (ii) a population-based sample of 259 Dutch children aged 12–13 years, pertaining to a case-control sampling frame of children with auditory verbal hallucinations. Multinomial regression analyses were carried out to investigate the associations between PE and ToM and HyperToM respectively. Analyses were adjusted for gender and proxy measures of general intelligence.

**Results:**

Low ToM score was significantly associated with PE in sample I (OR = 1.6 95%CI 1.1–2.3 χ^2^(4) = 12.42 p = 0.010), but not in sample II (OR = 0.9 95%CI 0.5–1.8 χ^2^(3) = 7.13 p = 0.816). HyperToM was significantly associated with PE both in sample I (OR = 1.8, 95%CI 1.2–2.7 χ^2^(3) = 10.11 p = 0.006) and II (OR = 4.6, 95%CI 1.3–16.2 χ^2^(2) = 7.56 p = 0.018). HyperToM was associated particularly with paranoid delusions in both sample I (OR = 2.0, 95%CI: 1.1–3.7% χ^2^(4) = 9.93 p = 0.021) and II (OR = 6.2 95%CI: 1.7–23.6% χ^2^(4) = 9.90 p = 0.044).

**Conclusion:**

Specific alterations in ToM may be associated with specific types of psychotic experiences. HyperToM may index risk for developing psychosis and paranoid delusions in particular.

## Introduction

A systematic review [1] documents that alterations in Theory-of-Mind (ToM) are found in people at familial risk of psychotic disorder. In addition, longitudinal studies propose that individuals who later develop schizophrenia, show ToM deficits in childhood [1]. Therefore, altered ToM may constitute an underlying, partly genetically mediated [1], [2], indicator of vulnerability for development of psychosis and schizophrenia [1], [3]. Recent studies in adult samples of both patients and individuals at familial risk of psychosis [4]–[9] have found altered ToM abilities to be associated not only with a diagnosis of schizophrenia but also, and more specifically, with specific psychotic symptoms [10]–[12]. However, these findings are diverging with regard to which ToM deficits are associated with which symptoms.

Hallucinations, delusions and other psychotic symptoms in the absence of diagnosable psychotic illness occur frequently in the general population. Recent meta-analyses found a median prevalence of such non-clinical Psychotic Experiences (PE) of around 7% in the general population of all ages [13], and around 17% in the subgroup of children aged 9–12 years [14]. PE are usually transitory, but longitudinal cohort studies have shown an association with later development of psychotic illness, indicating that PE may index psychometric risk for psychotic disorder [13]–[16].

PE and alterations in ToM are associated with each other [9], [17], but studies have for the most part been carried out in adult samples, and only three studies have examined these associations in non-clinical samples of children [18]–[20]. In two of the studies involving children, associations were found between current PE and poor ToM [18], [19]. In the third study, the overall score of ToM did not differentiate between children with and without PE [20]. However, in this study, lower ToM scores mediated the association between a lifetime history of auditory verbal hallucinations (AVH) and presence of delusions at age 12–13 years, i.e. children with AVH had a higher likelihood of forming secondary delusional ideation given lower ToM skills. This is in line with the other longitudinal study [18] finding that poor ToM at age 5 years predicted PE at age 12 years.

The divergence in findings regarding which symptoms of psychosis are related to ToM may be related to methodological dissimilarities across studies, e.g regarding the measurement of ToM and the classification of symptom clusters [1], [21], [22]. A more basic explanation may involve the heterogeneity and multidimensionality of ToM as a construct [23]. It has been suggested that deficits may occur in (i) the representational abilities (i.e. a conceptual deficit), so that an individual is unaware that others can hold a false belief about a state of affairs [24], [25], as well as in (ii) application abilities (i.e. deficit in performance), so that an individual has awareness of another person's mental life but fails to demonstrate or apply this knowledge due to processing constraints [26]. Frith (2004) suggested that schizophrenia patients with predominantly negative symptoms, disorganized behaviours, and early developmental problems lack representational abilities, whereas patients with paranoid symptoms would have the basic representational abilities but tend to excessively attribute intentions or self-referential meaning to others and therefore predict behaviour on the basis of these wrong beliefs [27]. This may be conceptualized as HyperToM, which refers to an exaggeration in the application of ToM [28]. Studies of both clinical [29] and general population [30] adult samples suggest that HypoToM is associated with a diagnosis of autism and negative symptoms [1], [29], [31], whereas HyperToM is associated with psychotic disorder [31] and psychotic symptoms [29], [32]. This type of erroneous ToM strategy appears to be specifically associated with delusions and particularly paranoid delusions comprising delusions of persecution, mind-reading and misinterpretation [29], [30], [32]. Thus, HyperToM may be a useful conceptual construct to improve the current insufficient differentiation between the various qualitative aspects of ToM [33] that may show different patterns of association with different developmental disorders [34].

While a limited number of studies on the presence of HyperToM in patients with psychosis have been carried out, no studies in at-risk individuals have systematically investigated this aspect of social cognition [33].

The aim of the present study was to examine the specific patterns of alterations in ToM (HyperToM) in relation to PE in children, in order to expand current knowledge on the role of ToM in the aetiology of psychosis.

### Research Hypotheses

We hypothesized that:

1. A lifetime history of PE would be more frequent in the group of children with overall ToM score at or below the median (Low ToM) compared to the group of children with ToM score above the median (High ToM) of the general population of children.
2. A lifetime history of PE would be particularly frequent in the group of children with the exaggerated type of ToM alteration (HyperToM) compared to children without this type of alteration.
3. The exaggerated type of ToM alteration (HyperToM) would be associated more strongly with a lifetime history of paranoid delusions (Pa) compared to a lifetime history of psychotic experiences without paranoid delusions (PE-NonPa).

## Methods

**Figure 1:**
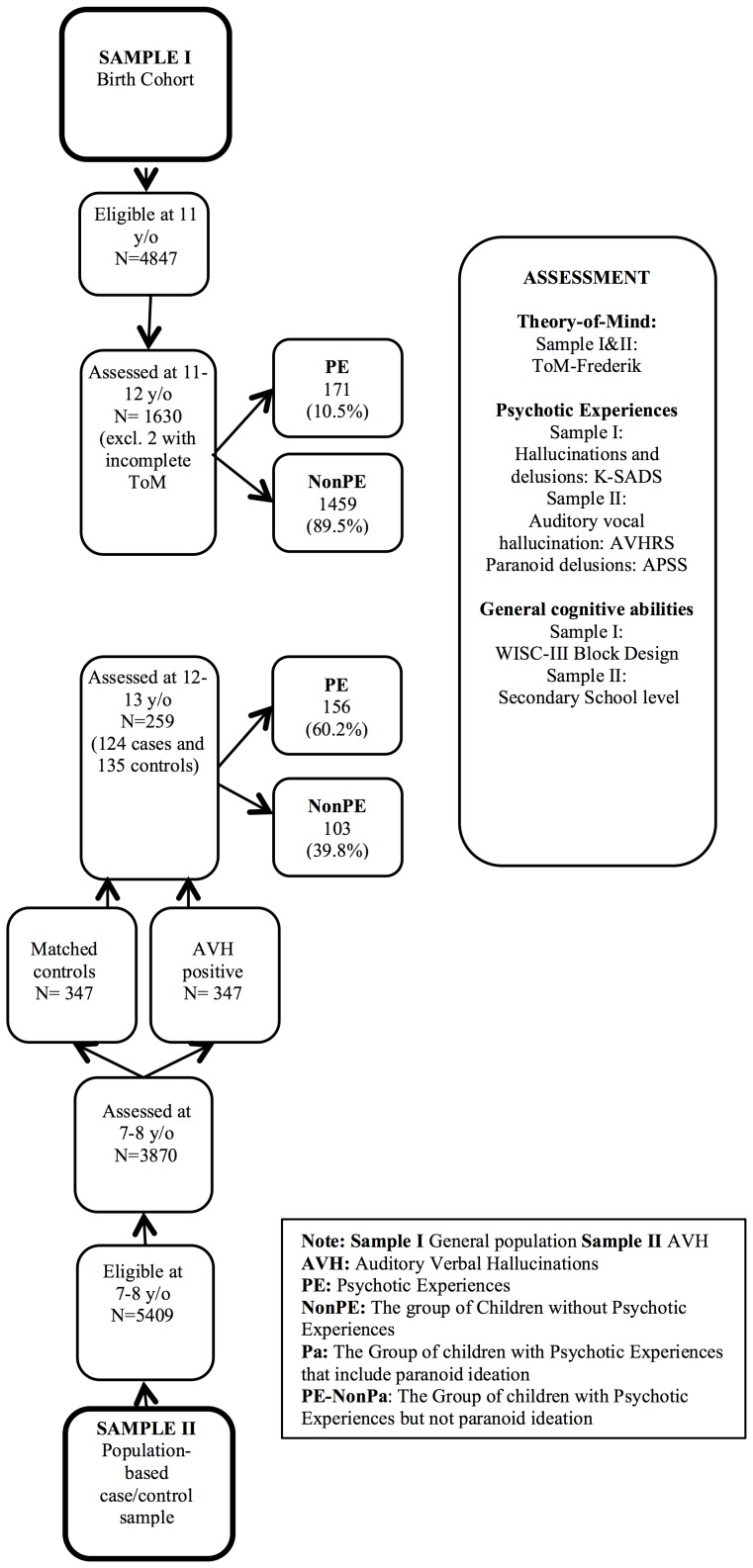
Overview of the study.

An overview of the study is presented in Figure 1.

The hypotheses were investigated in two studies covering two independent samples: (i) a general population sample of 1630 11–12-year-old Danish children [35] and (ii) a population-based sample of 259 12–13 year-old Dutch children, pertaining to a case-control sample of children aged 7–8 years when assessed for auditory verbal hallucinations (AVH) and 12–13 years when followed up for assessment of PE [20].

### Participants

#### Study I: The Danish general population sample of children aged 11–12 years

The current analysis was conducted as part of the 11–12-year follow-up of The Copenhagen Child Cohort 2000 (CCC2000). CCC2000 is a general population birth cohort, consisting of 6090 children born in 16 municipalities in the Copenhagen County in the year 2000, and followed prospectively since birth [36]. The cohort comprises 9% of all children born in Denmark that year, and is representative for children born in Denmark that year regarding key perinatal characteristics (Olsen, Skovgaard, Weile, & Jorgensen, 2007), except that ethnic minorities were somewhat overrepresented at baseline [36].

The study was conducted from May 1st, 2011 through October 1st, 2012. Cohort members were traced through The Danish Civil Registration System. 1.243 children were lost to follow-up (19 dead, 217 emigrated, 14 not traceable, and 993 whom had claimed ‘research protection’), leaving a total of 4847 children eligible for follow-up at age 11/12 years. The parents of eligible children were contacted by letter inviting them to sign up their child for a face-to-face assessment at the research clinic. Families living far away were offered home visits. Parents were contacted up to four times by letter and/or telephone. Gifts were offered in reward for participation. It was clearly stated that participation was voluntary and that data was kept confidential.

1632 children attended the assessment. Two of those had an incomplete ToM task, resulting in a final sample of 1630 children (34% of the eligible children, 48% boys). The mean age was 11.4 years. In a previous study of PE in the CCC2000 [35], the participating children were found to be slightly positively selected on a range of sociodemographic and perinatal background characteristics compared to the rest of the cohort (those alive at age 11 years).

#### Study II: Case-control sample of auditory verbal hallucinations

Sample II was recruited from a case-control sample of 694 children, half of whom had AVH when assessed at age 7 or 8 years as part of a population-based study of 3870 children. A total of 337 children from the case-control sample participated in the follow-up at age 12–13 years, in which 259 (49% of the eligible children, 47% boys) completed the ToM assessment (Bartels-Velthuis *et al.*, 2011b). Mean age was 13.1 years (SD 0.5). Socioeconomic status was evenly represented (31% low 38% middle, 31% high). The 78 children, in whom ToM was not assessed, more often were 13 as opposed to 12 years old (73% vs. 54% in the group with ToM assessment; (χ^2^ = 9.3, p = 0.002) and more often reported delusions (40% vs. 27% in the group with ToM assessment; χ^2^ = 4.3, p = 0.04). No other large or significant differences existed in demographic or outcome variables [20].

### Materials

#### Theory of Mind

The ToM Storybook Frederik (ToM-F) [37] was used to assess ToM in both studies. ToM-F is the Danish version of the ToM Storybook Frank [38] and is aimed at 10–14-year-old children and tests the understanding of second-order false beliefs, white lie, irony, double bluff and ‘faux pas’ (a socially awkward or tactless act). The children are asked a total of 24 questions while being presented with 16 pictures and listening to the storybook read aloud.

Originally, 32 questions were included but 8 questions where omitted in sample I. This reduction was done to adapt to the present study where the task was part of a larger assessment battery and thus needed to be shortened to keep the total time for the clinical assessment at a reasonable level, and minimize the strain on the children. The questions omitted were the ones producing the least differentiation between the children in a previous study [20]. To make direct comparison between sample I and II possible, analyses were carried out only on the 24 questions applied in both studies. As ToM scores on 8 questions from the original study thus were excluded from the data in sample II, ToM scores will not be identical to those reported in a previous publication [20] concerning sample II.

In both samples, the internal consistency between the 24 questions was moderate (sample I Cronbach's alpha = .69, sample II Cronbach's alpha = .64). The 24 questions consist of 16 ‘test’ and 8 ‘justification’ questions. The test answers are scored 0–1; 1 is given for a correct understanding of the situation (range 0–16). The 8 justification answers are classified according to 23 predefined categories (see “Appendix S1 Rating Categories for ToM Storybook” for full set of categories) [39] such as:

Desire: The answer refers to the protagonist's desire with respect to the situation. It involves wanting or desiring something.

Fact belief: The child refers to the protagonist's knowledge. It involves thinking, knowing, being sure of, expecting or recognizing.

Situational: Dwelling on the situation without reference to the mental state of the protagonist.

Based on these categories, the answers were scored (‘0’, ‘1’, ‘2’, and for some items ‘3’) on an ordinal scale predefined for each situation, resulting in a justification sum score (range 0–20), that was summed with the test score (‘0’ or ‘1’, range 0–16) to form the total ToM scale score of ToM abilities (range 0–36). Scores on the justification items depend on the level and quality of references made to the thoughts, beliefs, feelings or intentions of the story characters or the child itself.

The total ToM scale served as the primary measure of overall ToM abilities. The 8 justifications answers were also scored on a dichotomous scale as a non-mentalizing (‘0’) or mentalizing (‘1’) type of answer, regardless of the classification of the answer as formally right or wrong in the context, resulting in a mentalizing scale-score (range 0–8). Conform previous work, mentalizing answers were defined as those referring to beliefs, desires and similar inner states [30], [33]. Thus the following 11 of the 23 categories were considered mentalizing: Belief; desire; fact belief; value belief; mental state verb rest category; own frame of reference with mental state; emotion with empathy; emotion without empathy; perception with mental state; identification of first order intention; identification of second order intention (see Appendix S1 for a description of all categories). A high number of mentalizing answers (High-Mentalizing) was defined as scores above the median for population sample I.

From these scale scores we defined low ToM skills (Low ToM) as scores at or below the median on the total ToM scale. HyperToM was defined on the basis of (i) total ToM scale score below the median combined with (ii) number of mentalizing answers above the median in general population sample I. Both measures were taken into account, as the aim was to capture children that had a low level of overall ToM abilities while making frequent mental attributions. Taking both measures into account, a categorical variable was constructed, which captured children who were among the top 50% with regard to number of mentalizing answers, while at the same time among the lowest 50% with regard to overall ToM abilities measured by the total scale score These children showed a tendency to exaggerated and aberrant mentalizing explanations, labelled HyperToM, in contrast to the children without HyperToM. The phenotype should not be conceptualised as somewhere between high and low levels of overall ToM abilities, rather it is another dimension of ToM skills that was measured as a dichotomised variable. The median cut-offs from sample I (n = 1630) were also applied to assess HyperToM in sample II, thus providing the opportunity to replicate an association between this level of HyperToM and the presence of PE. The aim was to explore associations between the same quality of HyperToM, captured by this definition, and the presence of PE. Sample I was chosen to provide the level of cut-off, as this was the larger and most representative sample of children in the general population.

Ratings were carried out in close co-operation with the Dutch authors of *The ToM Storybook Frederik (ToM-F)*. An initial training seminar was conducted. Thereafter consensus ratings on a subsample of 200 children were carried out by a Danish rating group headed by the first author, and subsequently the remaining children were rated by the first author under supervision of the Dutch authors. A sample of 50 children was rated separately by one member of the Danish research group in order to measure the inter-rater reliability for assignment of children to each ToM group. The inter-rater reliability (computed as Cohen's Kappa) was very good for the categorizing of children as low ToM (kappa = 0.86) and as HyperToM (Kappa = 0.81) [44], [45].

#### Socioeconomic status

In Sample I **s**ocioeconomic status (SES) was derived from length of mothers' education and family disposable income. Low SES was set as low-skilled mother (length of education less than 10 years) and/or low family income (being in the lowest quartile of the entire cohort of 6090 children).

In Sample II SES was derived from parental averaged educational levels and family income, resulting in three levels: low (score = 1), middle (score = 2) and high (score = 3).

#### General Cognitive ability

As previous studies have pointed to the possibility that the association between schizophrenia and ToM performance may be confounded by general intelligence [1], [40], any study investigating cognitive mechanisms in psychosis should take the possible influence of general cognitive abilities into account [9]. Thus, measures of general cognitive ability were treated as a potential confounder of the association between ToM and PE in both studies.

In Sample I performance on the Block Design (BD) subtest of WISC-III was used as a proxy measure of children's general cognitive abilities. The BD subtest is highly correlated with full-scale IQ in the Wechsler intelligence test [41].

In Sample II secondary school level was categorized as follows: 1 (lower vocational) and 2 (higher/pre-academic) and was applied as a proxy measure of children's general cognitive abilities. This was not possible for 12 of the children, as 10 were still at primary school and data were not available for 2.

#### Psychotic Experiences

In Sample I PE were assessed in a semi-structured interview with K-SADS-PL [42], applying the screening section and the supplement on psychotic symptoms. Possible PE ratings were: absent, likely present, and definitively present. PE were rated present if at least one psychotic symptom was scored as ‘likely’ or ‘definitely’ present for two time periods: the past month and ‘lifetime before’. For the present study, a binary variable was created, coding “yes” or “no” PE for the lifetime period, ‘yes’ indicating that the child had experienced at least one psychotic symptom ‘likely’ or ‘definitely’ present in last month and/or in the lifetime before. The subgroup of children with delusional thoughts with paranoid content (Pa) were *a priori* defined as those with scores of likely or definitely present delusions of persecution, mind-reading and/or receiving messages from TV/radio, as these delusions have a clear paranoid content and can be reliably assessed in the general adolescent population [43]. Trained professionals including 2 psychologists, 3 medical doctors, 1 psychology graduate student and 1 medical student performed the interviews. All interviewers were trained by the senior researcher (PJ) and participated in regular training seminars supported by video-taped interviews, and including group discussions, ratings and supervision in order to obtain high inter-rater reliability in the observer-based ratings of the presence of psychotic symptoms, while taking into account attributions of symptoms to sleep, somatic disorders or toxic influences. If there was a clear attribution explaining the experience, the psychotic symptom was rated as not present. Based on 75 videotaped interviews, the inter-rater reliability was computed as Fleiss' Kappa for assignment of children to each group based on life-time presence of PE symptoms. The inter-rater reliability was very good for both the grouping into children with or without PE (Fleiss Kappa = 0.95) and the further grouping into 1) healthy children (NonPE) 2) children with Psychotic Experiences but no delusional thoughts with paranoid content (PE-NonPa) and 3) children with Psychotic Experiences that include delusional thoughts with paranoid content Pa (Fleiss Kappa = 0.85) [44], [45].

In Sample II PE were assessed using the screening question on AVH from the Auditory Vocal hallucination Rating Scale (AVHRS; [46]) and three items on delusional experiences from the Adolescent Psychotic Symptom Screener (APSS); [43]. The AVHRS is a structured 16-item interview evaluating experiences on AVH during a predetermined period (e.g. past month, past year). As opposed to the K-SADS applied in sample I, AVHRS only assess auditory Hallucinations. Each item consists of a compulsory question, followed by optional support questions. Items are scored on a 5-point scale, ordered in increasing severity, facilitating calculation of a severity score AVHRS [46]. The APSS items consist of questions on delusions (mind reading, persecutory delusions and receiving media messages). AVH were rated as: 0 (no) or 1 (yes) and were assessed at baseline over the past year and at follow-up over the five intervening years. Delusions were rated as: 0 (no), 1 (yes, likely) or 2 (yes, definitely) and were assessed over the lifetime [43]. A variable was constructed indicating presence of at least one (definite) delusion (hereafter: delusions). As in sample I, Pa was defined as any delusion of persecution, mind-reading and/or receiving messages from TV/radio. Based on the 337 children attending this part of the assessment, interrater reliability for AVH was good (kappa = 0.88).

### Statistical analysis

Analyses were carried out using Stata 12 software (StataCorp LP, College Station, TX). Descriptive statistics were used to analyse sample characteristics. Group differences on demographic variables, ToM scores as well as the proxy measures of general cognitive ability were tested by chi-squared tests for categorical variables and by independent samples t-tests for continuous variables.

Logistic regression analyses were conducted with HyperToM, ToM total score, low mentalizing, gender and the proxy measures of general cognitive ability (BD respectively “school level”) as independent variables. In each logistic regression analysis, the predictive effect of each of the ToM scale scores was estimated while adjusting for the proxy measures of general intelligence.

The logistic regression analyses were used to estimate 1) the risk of having a lifetime presence of PE (yes/no) for the group of children with low ToM compared to the group of children with high ToM in the two samples; and 2) the risk of a lifetime presence of PE (yes/no) for the group of children with HyperToM compared to those children without HyperToM in the two samples. The relative risk was calculated as odds ratio (OR) with 95% CI.

Finally, multinomial logistic regression analyses were used to estimate the dose-response relationship between HyperToM (yes/no) and having a lifetime history of paranoid delusions (Pa), a lifetime history of psychotic symptoms without paranoid delusions (PE-NonPa), and no lifetime history of PE. The hypothesis being tested was that the risk for Pa versus no PE when exposed to HyperToM would be higher than the risk for PE-NonPa versus no PE when exposed to HyperToM.

Thus, the estimated ORs express the risk of experiencing Pa relatively to no PE, and the risk of experiencing PE-NonPa relatively to no PE for children with HyperToM compared to the children without HyperToM. All analyses of risk of HyperToM status were repeated substituting HyperToM as predictor with each of the underlying dimensions of HyperToM, i.e. low ToM score and high mentalizing answers in order to be able to compare the OR of HyperToM to the OR of these variables. All multinomial logistic regression analyses were adjusted for gender. Post-estimation analysis was carried out to examine if the estimated OR of HyperToM for Pa was significantly different from the OR of HyperToM for PE-NonPa.

### Ethics

The Danish study (sample 1) was approved by The Danish Data Protection Agency (J.nr.2010-41-4438), the Capital Region of Denmark (J.nr. 2007-58-0015), and The National Committee on Health Research Ethics was consulted (J.nr.H-C-FSP-2010) in accordance with national guidelines. The written information for sample 1 clearly stated that 1) study participation is voluntary 2) consent can be withdrawn at any time and 3) all data will be de-identified. Parents and child agreed to participate by signing up for the face-to-face assessment of child at the hospital. This consent procedure was approved by the ethics committee.

In the Dutch study (sample 2) written informed consent was obtained from both parents and children, as approved by the local ethics committee.

## Results

### Sample 1

#### Psychotic Experiences

A total of 171 (10.5%) of the 1630 children reported lifetime PE. The further subgrouping of these 171 children based on the presence or absence of Pa yielded a Pa group of 70 (41%) children, and a PE-NonPa group of 101 (59%) children (Table 1). There were no significant differences regarding age or gender between either the PE and Non-PE groups, or between the Pa and PE-NonPa groups (Table 2).

**Table 1:** Demographic, ToM and PE data from sample I and sample II.

	Sample I	Sample II
**N**	1630	259
**Age in months**	137	159
**Gender (% boys)**	782 (48)	122 (47)
**Low Socioeconomic status N(%)**	382 (23)	80 (31)
**General cognition**		
Block Design (M, SD)	9.7 (3.9)	-
School Level - Lower vocational (N, %)	-	135 (55)
**ToM score**		
Mean (SD)	19.2 (4.2)	21.9 (4.6)
Range	3–33	12–33
Median	19	21
**Mentalizing answers**		
Mean (SD)	3.3 (1.5)	3.8 (1.7)
Range	0–8	0–8
Median	3	3
**HyperToM**	202 (12%)	21 (8%)
**Psychotic Experiences (PE)**	171 (10.5%)	156 (60%)
Hallucinations	68 (40%)	85 (55%)
Delusions	26 (15%)	22 (14%)
Both	77 (45%)	49 (31%)
**Psychotic Experiences**		
PE-NonPa	101 (59%)	85 (54%)
Pa	70 (41%)	71(46%)

**Table 2:** Between-group comparisons for differences in age, gender distribution and measures of cognition in children with psychotic experiences compared to children without and in children with paranoid delusions compared to children with psychotic experiences but without paranoid delusions.

	PE subsamples				Pa Subsamples			
	PE	Non-PE	Analysis	p	Pa	PE-NonPa	Analysis	p
**SAMPLE I**	N = 171	N = 1459			N = 70	N = 101		
Gender (N, %boys)	77 (45)	715(49)	χ^2^(1) = 0.7794	0.38	30 (43)	47(47)	χ^2^(1) = 0.2259	0.635
Age in months (Mean, SD)	139 (4.7)	139 (4.7)	t (1628) = 1.04	0.29	140.1 (4.6)	139.8 (4.8)	t(169) = −0.3409	0.734
Block Design (Mean, SD)	9.3 (3.9)	9.8 (3.8)	t(1628) = 0.14	0.13	9.1 (3.9)	9.5 (3.9)	t(169) = 0.6346	0.527
**SAMPLE II**	N = 156	N = 103			N = 71	N = 85		
Gender (N, %boys)	73 (47)	47 (46)	χ^2^ (1) = 0.0812	0.776	40 (56)	42 (49)	χ^2^(1) = 0.7443	0.388
Age in months (Mean, SD)	159 (5.9)	160 (5.8)	t (257) = 1.7254	0.09	159.4 (5.7)	158.4 (6.0)	t(154) = −1.0171	0.311
School(N, %preacademic)*	81 (52)	59 (57)	χ^2^(2) = 2.0765	0.354	33 (46)	54 (64)	χ^2^(1) = 4.7688	0.029

*missing data on 12 children.

#### Theory-of-Mind Frederik

The median ToM score was 19 and the median for number of mentalizing answers was 3 (Table 1). Based on these numbers, a total of 827 (51%) were classified as having low ToM, and among these 202 (12%) children were classified as having HyperToM. There were no significant differences in neither gender (47% vs 48% boys χ^2^(1) = 0.1310, p = 0.717) nor age (139 vs 139 months t(1628) = 1.0381, p = 0.2994) between the groups with and without HyperToM.

#### Proxy General Intelligence: Block Design

There were no significant differences on BD score between the PE and NonPE groups, and similarly no differences between the Pa and PE-NonPa groups. The group of children with high ToM had a significantly higher BD Score than the children with low ToM (Table 2). The HyperToM group (mean = 9.1, SD = 3.7) had a significantly lower BD score than the Non-HyperToM group (mean = 9.8, SD = 3.8, t(1628) = 2.6978, p = 0.007). All subsequent analyses were adjusted for BD score.

#### Theory-of-Mind and Psychotic Experiences

The children with a low ToM score had an increased risk of having PE compared to the children with high ToM score (adjusted OR = 1.6, 95%CI 1.1–2.3, χ^2^(4) = 12.42 p = 0.010). The children with HyperToM compared to the children with no HyperToM also had an increased risk of PE (adjusted OR = 1.8, 95%CI: 1.2–2.7 χ^2^(3) = 10.11) p = 0.006). The estimated risks of PE for poor ToM and for HyperToM were within the same range with almost identical 95% CIs (Table 3).

**Table 3:** Differences in frequency of Psychotic Experiences by ToM groups (and gender and measures of general cognition).

SAMPLE I	Odds Ratio for having PE			
	OR	95% CI	X^2^	p
**MODEL 1**				
Gender	1.2	0.8–1.6	X^2^(4) = 12.42	0.361
Block Design	0.9	0.9–1.0	X^2^(4) = 12.42	0.254
**ToM Scores**				
High Number of Mentalizing answers	1.6	1.1–2.6	X^2^(4) = 12.42	0.011
Low ToM Score	1.6	1.1–2.3	X^2^(4) = 12.42	0.010
**MODEL 2**				
Gender	1.2	0.8–1.6	X^2^(3) = 10.11	0.347
Block Design	0.9	0.9–1.0	X^2^(3) = 10.11	0.165
HyperToM	1.8	1.2–2.7	X^2^(3) = 10.11	0.006

SAMPLE II	Odds Ratio for having PE			
	OR	95%CI	X^2^	p
**MODEL 1**				
Gender	1.1	0.7–1.8	X^2^ (3) = 7.13	0.755
**ToM Scores**				
High Number of Mentalizing answers	0.5	0.3–0.9	X^2^(3) = 7.13	0.021
Low ToM Score	0.9	0.5–1.8	X^2(^(3) = 7.13	0.816
**MODEL 2**				
Gender	0.8	0.5–1.4	X^2^ (2) = 7.56	0.479
HyperToM	4.6	1.3–16.2	X^2^(2) = 7.56	0.018

The adjusted OR for Pa in the HyperToM group versus the group without HyperToM was 2.0 (95%CI: 1.1–3.7 χ^2^(4) = 9.93 p = 0.021), whereas the adjusted OR for having PE-NonPa was non-significant albeit directionally similar (Table 4). Post-estimation analysis confirmed that the OR for the children with HyperToM compared to those without HyperToM was significantly higher for Pa than for PE-NonPa (χ^2^ (2) = 7.96, p = 0.0187).

**Table 4:** Differences in frequency of Psychotic Experiences without paranoid delusions and Psychotic Experiences with Paranoid delusions by ToM groups, gender and measures of general cognition.

STUDY	PE-NonPa				Pa			
N = 1630	OR	95% CI	X^2^	p	OR	95% CI	X^2^	p
**MODEL 1**								
**Gender**	1.1	0.7–1.6	X^2^(8) = 17.51	0.707	1.3	0.8–2.1	X^2^(8) = 17.51	0.307
**Block Design**	0.9	0.9–1.0	X^2^(8) = 17.51	0.716	0.9	0.9–1.0	X^2^(8) = 17.51	0.160
**ToM Scores**								
High number of mentalizing answers	1.2	0.8–1.9	X^2^(8) = 17.51	0.387	2.3	1.4–4.0	X^2^(8) = 17.51	0.002
Low ToM score	1.6	1.0–2.6	X^2^(8) = 17.51	0.038	1.6	0.9–2.7	X^2^(8) = 17.51	0.105
**MODEL 2**								
**Block Design**	0.9	0.9–1.0)	X^2^(4) = 9.93	0.504	0.9	0.9–1.0	X^2^(4) = 9.93	0.179
**HyperToM**	1.6	0.9–2.8	X^2^(4) = 9.93	0.073	2.0	1.1–3.7	X^2^f(4) = 9.93	0.021

STUDY II	PE-NonPa				Pa			
N = 259	OR	95% CI	X^2^	p	OR	95% CI	X^2^	p
**MODEL 1**								
**Gender**	0.8	0.5–1.5	X^2^(6) = 12.75	0.524	1.1	0.6–1.9	X^2^(6) = 12.75	0.874
**ToM Scores**								
High number of mentalizing answers	2.6	1.2–5.4	X^2^(6) = 12.75	0.012	1.6	0.8–3.4	X^2^(6) = 12.75	0.204
Low ToM Total score	0.9	0.4–1.9	X^2^(6) = 12.75	0.755	1.3	0.6–2.8	X^2^(6) = 12.75	0.481
**MODEL 2**								
**Gender**	0.8	0.4–1.4	X^2^(4) = 9.90	0.365	0.9	0.5–1.7	X^2^(4) = 9.90	0.816
**HyperToM**	3.3	0.8–13.1	X^2^(4) = 9.90	0.098	6.2	1.7–23.6	X^2^(4) = 9.90	0.044

### Sample II

#### Psychotic Experiences

Of the 259 children in the case-control follow-up sample, 156 (60.2%) reported lifetime PE. The further subgrouping of these 156 yielded a Pa group of 71 children (46%) and a PE-NonPa group of 85 children (54%). Similar to sample I there were no significant differences in either age or gender between either the PE and Non-PE groups, or between the Pa and PE-NonPa groups (Table 2).

#### Theory of Mind Frederik

The median ToM score was 21, and the median number of mentalizing answers was 3 (Table 1). Applying the same criteria for HyperToM as in sample I (i.e. cut-offs: low ToM = ToM total score <19, and High mentalizing = number of mentalizing answers >3), 21 (8%) of the children in sample II were classified as having HyperToM. A significantly greater part of the group of children with HyperToM (81%) were girls compared to the group of children without HyperToM (51% χ^2^(1) = 7.0293 p = 0.008). There were no age-differences between the group with HyperToM (159 months) and without HyperToM (159 months t(257) = 0.5699 p = 0.5692).

#### Proxy General Intelligence: School level

Among the 247 children with existing data at school level, there were no significant differences in the percentages of the children attending pre-academic school in the PE compared to the NonPE group. However, significantly fewer children in the group of children with Pa (46%) compared to the group of children with PE-NonPa (64% χ^2^(1) = 4.7688 p = 0.029) (table 2). Significantly fewer children attended pre-academic education in (i) the low ToM (37%) compared to the High ToM group (64% χ^2^(1) = 17.3139 p<0.001) and in (ii) the HyperToM (28%) compared to the non-HyperToM group (57%, χ^2^(1) = 5.6594 p = 0.017).

To avoid having to exclude from the analyses the 12 children, of whom 8 had PE but missing data at school level, we first carried out both adjusted and non-adjusted analyses for the smaller sample of 247 children. As none of these analyses differed substantially in terms of estimated effects or level of significance, non-adjusted analyses were reported thus allowing inclusion of the full sample of 259.

#### Theory-of-Mind and Psychotic Experiences

The children with low ToM did not have a significantly increased risk of having PE compared to the children with high ToM. The children with HyperToM had an increased risk of PE compared to the children without HyperToM (OR = 4.6, 95%CI 1.3–16.2, χ^2^ = (2)7.56 p = 0.018) (Table 3). The OR for Pa in the HyperToM group versus the group without HyperToM was 6.2 (95%CI 1.7–23., p = 0.002), whereas the OR for having PE-NonPa was non-significant albeit directionally similar (Table 4). Post-estimation analysis confirmed that the OR for HyperToM was significantly greater for Pa than PE-NonPa (χ^2^ (2) = 7.96, p = 0.018).

## Discussion

### Findings

The main finding of the present study was that HyperToM was associated more strongly with Pa than PE-NonPa. Confirming hypothesis 3, the OR was 2.0 for Pa in sample I and 1.6 for PE-NonPa, and in sample II the OR for Pa was 6.2 and 3.3 for PE-NonPa. Thus the study documents a clear, replicated relationship between an exaggerated theory of mind and paranoid symptoms in childhood.

Our first hypothesis on PE being more frequent in children with low ToM was confirmed in sample I as the odds for having PE was 1.6 times greater in the group of children with a low ToM score than in the group of children with a high ToM score. However, this was not the case in sample II, where no significant association was apparent.

Hypothesis two on PE being particularly frequent in children with HyperToM was confirmed, as the OR for having PE in both samples was higher for the HyperToM group than for the group without HyperToM. In sample I, the odds for PE was 1.8 times greater in the HyperToM group versus the Non-HyperToM group. In sample II, the corresponding estimate was a 4.6 times greater risk for PE in the HyperToM group versus the non-HyperToM group, while the risk of PE showed no significant association with poor ToM abilities measured as an overall low ToM score. Only in sample I, the odds of PE were within the same range, and with overlapping CIs, for children with poor ToM and for children with HyperToM.

### ToM and development of psychosis

The results suggest that particularly the HyperToM type is associated with PE, and that this association is strongest for delusional ideas with paranoid content (Pa), i.e, delusions of persecution, mind-reading and/or receiving messages from TV/radio. These findings are in line with theories suggesting that while general alterations in ToM may be a vulnerability marker for psychosis, more specific types of alterations [29], [47] may have a mediating role in the development of different types of specific symptoms.

Based on the work of Frith [48], it may be argued that development of ToM abilities, including the awareness of intentions of the self and others, is necessary in order to experience ideas of reference and symptoms like mind reading. Clearly, paranoid patients have intact ToM in the sense that they know that other people have mental states, but may perform poorly because of their difficulties in accurately monitoring other people's intentions [40], [49].

HyperToM may in part be stress-induced. Increased stress responsiveness affects mentalizing capacity [50]. For example, a recent study [51] found that high cortisol-responding women make more mentalizing errors due to a tendency to develop HyperToM after stress induction, thus demonstrating that the environment, in the context of increased stress responsiveness, modulates the functioning of ToM abilities. Frith [4], [48] suggested that whereas paranoid patients may have a normally functioning ToM when asymptomatic, during acute illness, reduced ToM functioning may lead to the belief that others have malevolent intentions [21]. Such combinations of trait and state-like qualities have been reported for other meta-cognitive mechanisms of psychotic symptoms such as a jumping-to-conclusions reasoning style [52].

Due to the cross-sectional design of the present study, etiological issues cannot be addressed. Future studies may examine how HyperToM develops, and how it may be related to maturational processes. HyperToM may be related to the inability to take perspectives as suggested by Langdon and colleagues [53] or to a tendency to overgeneralization of hypotheses, [29], [32], [34], [54], but these theories have yet to be tested in longitudinal studies.

A retrospective study of young patients with schizophrenia [55] found an increased likelihood of delusional beliefs with age, indicating that the formation of these symptoms from preadolescence onwards depend on brain maturation and learning. Similarly, HyperToM may also depend on brain maturation and learning [56] The immaturity of the more advanced aspects of ToM abilities in preadolescent children may increase the likelihood of social misinterpretation, anxiety and formation of paranoid and first-rank like delusional ideation that may ultimately result in the persistence of PE [55].

### Strengths and limitations

A main strength of the study is the fact that the hypotheses were tested in two large, independent population-based samples from two different countries. PE and ToM were assessed using the same items and cut-offs. The age range was fixed around the onset of puberty and was roughly the same in the two samples. Mean age had no influence on any analysis.

There are, however, a few limitations that need to be considered. First, we were not able to control for general cognition with a measure of full IQ, but adjustment for appropriate proxy measures of IQ did not substantially change any of the results. Secondly, the psychometric properties of the ToM-F task have not yet been examined extensively. Nevertheless, the internal consistency of the task was found to be fair and comparable across the samples, and the scores were close in range. Also, ToM-F is based on the ToM Storybooks for younger children, which have good psychometric qualities [39].

Finally, the cross-sectional design does not make it possible to determine the direction of the relationships. Longitudinal studies need to further explore the developmental trajectories of HyperToM in childhood and adolescence and to assess both the causal and temporal relationships between PE and HyperToM, and the stability of HyperToM over time.

### Conclusion

The current study is the first to demonstrate patterns of association between specific alterations in ToM and psychotic symptoms in a population-based sample of children. The findings were replicated in a smaller, high prevalence study.

Our findings suggest that it may be productive to apply more differentiated measures of ToM instead of relying on an overall score based on number of correct answers. This could be helpful not only when conducting research on the development of PE and psychosis, but also when applying ToM tasks as part of a clinical assessment in child mental health service settings.

The results contribute to the understanding of the relationship between ToM and psychosis, which seems unlikely to be fully explained by an association between poor ToM and a state of psychosis. The current study points to the possibility of different alterations in ToM impacting on different stages of development of psychosis.

## Supporting Information

Appendix S1 **Rating Categories for ToM Storybook.**
(DOCX)
