# Supplementary material for: Hyper-Theory-of-Mind in Children with Psychotic Experiences
Source: PLoS One. 2014 Nov 14;9(11):e113082. doi: 10.1371/journal.pone.0113082 (PMC4232592; doi:10.1371/journal.pone.0113082)
Supplement: Appendix S1 — Rating Categories for ToM Storybook. (DOCX) [file pone.0113082.s001.docx]

**APPENDIX 1 – RATING CATEGORIES FOR ToM**

**Reiteration of question**: When the answer is a repetition of an emotion or action from the question. This does not have to be a literal repetition.

**Verb referring to a belief**: Answers in which the verb “say” (or “tell”) is used instead of “think”. It is understood that saying is like thinking aloud and thus indicates belief. This only applies when it is explicitly mentioned in the text, that the protagonist thinks.

Remarks:

When ”say” is explicit in the text then it should not be rated as belief, but as ‘Situational’*.*

If ”say” is used in the literal sense, but combined with a perception term, then it should be rated as ‘Perception criterion + Perception criterion

**Desire**: The answer refers to the protagonist’s desire with respect to the situation. It involves wanting or desiring something.

**Fact belief**: The child refers to the protagonist’s knowledge. It involves thinking, knowing, being sure of, expecting or recognizing.

**Value belief**: Answers to these questions reflect a value judgment on how the protagonist handles a situation. It involves verbs such as loves, dares, liking something, or finding it sad.

**Situational**: Dwelling on the situation or illustration without reference to the mental state (desire/belief) of the protagonist.

**Location possession explanation**: The child very clearly refers to the location or someone’s possession of an object (as specified in the question), without referring to the mental state of the protagonist.

**Perception criterion**: The child refers to a reality criterion: the use of senses (hearing, seeing, smelling, tasting, feeling or derivatives) by the protagonist.

**Mental state-verbs not otherwise specified**: These constitute of verbs referring to mental states, but do not fall under categories ‘desire’, ‘fact belief’, ‘value belief’, or ‘verbs which refer to a belief’. They are: counting on, being afraid that, finding sad that, worrying about, looking forward to, being happy with, being anxious about, hoping for, liking, pitying that, being curious about, wondering about, must, may, having intention to, planning, is going to, being honest about, betraying, trusting, believing.

**Own reference frame with mental state**: In these situations the child describes a mental state, giving an answer in the form of a belief or desire (think, know, like, want, dare, etc.), or that an emotion is involved in the answer. However, this answer refers to the child himself; how he/she would react in the same situation. Or the child gives an own interpretation of the situation and makes up things that (indirectly) relate to the context of the question, but goes too far.

**Own reference frame without mental state**: This answer is similar to the former one, but without using a mental state. The answer does not contain a belief, desire, emotion or other mental state expression.

**General knowledge reference:** The child refers explicitly to a normality or logicality.

**Emotions with empathy**: The child gives an answer showing that the protagonist refers to the emotions of one or more other persons and also names these others persons. Examples are: cheerful, happy, disappointed, hurt, comfortable, bewildered, sad, scared, angry, uncertain, and horrified.

**Emotions without empathy**: The child gives an answer showing that the protagonist refers to his/her own emotions without referring to the emotions of one or more of the other persons. Examples are: cheerful, happy, disappointed, hurt, comfortable, disappointed, sad, scared, angry, uncertain, and horrified.

**Simple emotion justification**: This category is only applied in the 7^th^ justification question. In this question the child is explicitly asked to describe the mood of one of the children in the story. There is a distinction between Emotions without empathy and Simple emotion justification. The difference is that the emotions classified as Simple emotion justification more effectively describes how the child feels.

**Perception criterion + Perception criterion**: The child refers to a perception twice. This also includes answers where the child, parallel with reference to the protagonist's use of the senses, uses the word ”say” or a derivative thereof (said, spoke). In this case ”say” is also categorized as a Perception criterion, and constitutes an example of two perception criterions in one justification question.

**Perception criterion with mental state**: The child refers to a mental state (belief or desire) and besides refers in one of the following ways to a perception criterion:

- The child points out the protagonist's use of the senses.
- The child refers to ”say” or a derivation thereof (said, spoke).

**Belief + desire**: The child gives an answer that refers to both a "desire" and a “fact belief”.

**Belief + belief**: The child gives an answer that refers to a “fact belief” twice .

**Identification of the speaker’s first-order intention**: The answer shows that the protagonist intends to influence the behavior of one or more others.

**Identification of the speaker’s second-order intention**: The answer shows that the protagonist intends to influence the mental state of one or more others.

**Identification of an attitude**: The answer refers either to the attitude of the protagonist or a statement regarding the situation, or to functions such as irony or sarcasm. But the answer does not show that the protagonist intends to influence the behavior or mental state of the other.

**Irrelevant/uninterpretable**: The answer is a nonsense answer; it has nothing to do with the question and is thus neither an explanation nor an answer to the question.

**Does not know**: When a child says he/she does not know the answer.

**Does not say**: When a child remains silent; he/she gives no answer.

**Missing**: The answer is not given, unreadable or inaudible.

**Not applicable**: When a question was accidently not asked.
